# The Story of the Insane from Year to Year

**Published:** 1889-09-28

**Authors:** 


### The Story of the Insane from Year to Year.

Asylum Reports.

### JOINT COUNTIES' ASYLUM, CARMARTHEN.

In this asylum are the patients from Cardiganshire, Carmarthenshire, and Pembrokeshire. They now number 529, being an increase of 21 during the year. There were 87 admissions, 25 discharges, and 41 deaths. In 26 cases the insanity was hereditary, and in 11 it was owing to intemperance. It would seem that the proportion of hereditary insanity is much higher in these counties than in England and Wales generally, and this may be owing to the comparative absence of migration and emigration, necessitating frequent marriages between relations. The recoveries stand at 23·3 per cent., and deaths at 7·93 per cent. Patients who are suffering from a first attack of insanity of not more than three months' duration Dr. Hearder places in the first class as regards chances of recovery, and he finds that only 17 of the year's admissions belong to this class. This is very lamentable when it is remembered that medical superintendents are always urging the authorities to send cases of insanity early to the asylum. Hereditary predisposition acounts for 26 of the admissions, and drink for 11. Ten of the deaths were owing to phthisis. The weekly cost of maintenance is 7s. 5d.

### SURREY COUNTY ASYLUM AT BROOKWOOD.

This asylum is stated to have been practically full during the whole of the year, and on December 31 it contained 1,052 patients. There were 226 admissions, 118 discharges, and 102 deaths. The recoveries were 36·66 per cent., and the deaths 9·61 per cent. Thirteen of the deaths were caused by phthisis; and there were six cases of typhoid fever, but only one died, and in this the disease was complicated with pneumonia. Dr. Barton says: "The admissions were of a much more unfavourable type than those of the previous year, and a large proportion were suffering from organic brain disease. No doubt the continual depression in trade and agriculture influenced to a great extent the character of the insanity. There was a marked decrease in the acute and uncomplicated forms from which the greater proportion of the curative class is drawn, and a corresponding increase in the number of melancholics and dements." We learn from the report of the visitors that the head male attendant and four of the subordinate staff received pensions during the year. The former received the maximum pension allowed by the Lunacy Acts—namely, two-thirds of salary and allowances. The weekly rate of maintenance is nearly 10s.

### ENNIS DISTRICT ASYLUM.

On January 1, 1888, the numbers were 350, and on December 31 they had increased to 358. During the year 75 patients were admitted, 56 were discharged, and 11 died. The recoveries stand at 53·3 on the admissions, and the deaths at 3 per cent. on the average number resident. The recoveries are above the average, and the deaths are under the average of asylums generally, and of this asylum in former years. The male side of the asylum is quite full, but there are 21 vacancies on the female side. Dr. Gelston foreshadows the enlargement of the building, and it would seem that the want of accommodation for the insane is felt in various parts of Ireland, although the population of the country is diminishing. We learn from Dr. Gelston's report that "a very determined attempt was made to burn down the asylum on the morning of the 12th August last, a male patient, who had been for some months employed daily in cleaning the house, having set fire to no less than eight beds in two of the dormitories. Fortunately, the fire was discovered immediately and extinguished." In twelve of the admissions the disease was caused by intemperance, and in the same number it was hereditary. The visitors do not publish an annual report.

### WEST RIDING ASYLUM, WAKEFIELD.

This asylum contained 1,354 patients at the end of the year. The admissions were 455, the discharges 338, and the deaths 165. The recoveries stand at 33·18 per cent., and the deaths at 11·75. The percentages of recoveries and deaths in this asylum are known from the year 1818, and we notice that the former has an average of 42·35, and the latter 13·21 for the seventy-one years. Of the deaths during 1888, 42 were from general paralysis, 27 from phthisis, and one from enteric fever. In 157 of the admissions, the cause of the insanity was hereditary influence; and in 90, it was drink. For upwards of twenty years this asylum has been noted for the amount of purely medical work done in the wards, and there would seem to be no falling off in this feature which has made Wakefield famous. Dr. Lewis says: "It is with great pleasure I have to record the continued interest taken in pathological work by my colleagues generally; the advantage taken of the material and appliances at their disposal in the laboratory provided for this purpose by the committee; and the ability and unwearied energy displayed by Dr. Bullen in this his special department."

### NOTTINGHAM BOROUGH ASYLUM.

There is proper space in this asylum for 292 patients, but on December 31, 1888, it contained 311, and in addition to this there were 85 cases boarded out in other asylums. The average annual increase of patients is something like three per cent. on the average number resident; and although this is a perfectly well-known and recognised fact, it is strange how often it is ignored by committees, with the invariable result that the boarding-out process has to be adopted. This means that patients are sent away and their friends find it difficult to visit them; they are, probably, not seen except at long intervals by their own magistrates, and the ratepayers has to find the extra cost of maintenance—thirteen or fourteen shillings weekly, instead of eight shillings, per patient. The admissions amounted to 104, the discharges 54, and the deaths to 41. The percentage of recoveries would come out at 28·8 on the admissions, and the deaths at 13·4 on the average number resident. It should be said that the average of the former during the eight years of the asylum's existence is 41·4 and the latter stands at 10·7. As usual, the chief causes of insanity were drink and hereditary influence. Not fewer than 23 of the admissions were suffering from general paralysis, 4 were epileptics, 15 were chronic maniacs, 21 were chronic melancholics, and 12 were demented cases or cases suffering from cerebral disorganisation. This would show 75 incurable cases out of 103, and yet some people actually tell us that 60 per cent. would be cured if we had hospitals for recent cases.

